# Impact of Hot Environment on Fluid and Electrolyte Imbalance, Renal Damage, Hemolysis, and Immune Activation Postmarathon

**DOI:** 10.1155/2017/9824192

**Published:** 2017-12-21

**Authors:** Rodrigo Assunção Oliveira, Ana Paula Rennó Sierra, Marino Benetti, Nabil Ghorayeb, Carlos A. Sierra, Maria Augusta Peduti Dal Molin Kiss, Maria Fernanda Cury-Boaventura

**Affiliations:** ^1^Institute of Physical Activity and Sports Sciences, University of Cruzeiro do Sul, São Paulo, SP, Brazil; ^2^School of Physical Education and Sport, University of São Paulo, São Paulo, SP, Brazil; ^3^Sports Cardiology Department, Dante Pazzanese Institute of Cardiology, São Paulo, SP, Brazil

## Abstract

Previous studies have demonstrated the physiological changes induced by exercise exposure in hot environments. We investigated the hematological and oxidative changes and tissue damage induced by marathon race in different thermal conditions. Twenty-six male runners completed the São Paulo International Marathon both in hot environment (HE) and in temperate environment (TE). Blood and urine samples were collected 1 day before, immediately after, 1 day after, and 3 days after the marathon to analyze the hematological parameters, electrolytes, markers of tissue damage, and oxidative status. In both environments, the marathon race promotes fluid and electrolyte imbalance, hemolysis, oxidative stress, immune activation, and tissue damage. The marathon runner's performance was approximately 13.5% lower in HE compared to TE; however, in HE, our results demonstrated more pronounced fluid and electrolyte imbalance, renal damage, hemolysis, and immune activation. Moreover, oxidative stress induced by marathon in HE is presumed to be related to protein/purine oxidation instead of other oxidative sources. Fluid and electrolyte imbalance and protein/purine oxidation may be important factors responsible for hemolysis, renal damage, immune activation, and impaired performance after long-term exercise in HE. Nonetheless, we suggested that the impairment on performance in HE was not associated to the muscle damage and lipoperoxidation.

## 1. Introduction

Thermoregulation processes maintain the body temperature in a physiological range despite elevated metabolic rates and exposure to hot environments (>30°C); however, marathon running performance decreased by 2–10% approximately as the wet bulb globe temperature (WBGT) increased from 10 to 25°C [1]. The air temperature is the chief weather parameter that correlates to completing the time anomaly and percentage of runners who do not complete the marathon race [2].

Previous studies have demonstrated the physiological changes induced by exercise exposure in hot environments [3, 4]; however, the mechanisms involved in the impaired aerobic exercise performance in warm–hot conditions remain unclear [3–5]. Hypohydration (body water deficit of >2% of body mass) caused by excessive sweat loss begins to impair the aerobic performance when the skin temperatures exceed 27°C and has pronounced influence on impairing the aerobic performance in warm–hot environments [3, 4].

However, the impairment of performance induced by prolonged exercise in hot environments, with or without dehydration (>2% body mass), is associated to one or more alterations in the hematological parameters, redox balance, tissue damage, skeletal muscle metabolism, and cardiovascular system [5].

Endurance exercise in hot environment induces pronounced catecholamine and cortisol, with proinflammatory and compensatory anti-inflammatory responses, which may influence the tissue damage [6–8]. The environmental heat stress also promotes blood flow and oxygen limitations to the skeletal muscle implicating the impairment of VO_2max_ modifying oxygen metabolism and oxidative stress [5].

Oxidative stress is caused by an imbalance between the generation of reactive oxygen species (ROS) and their clearance by the antioxidant defense system. Endogenous sources of ROS include the mitochondrial respiratory chain, peroxisomes, NADPH oxidase, hemoglobin, myoglobin, and catecholamine autoxidation during exercise [9]. The excessive production of ROS in the hot environment may lead to the oxidative damage of macromolecules, immune dysfunction, and muscle damage. Recently, Mrakic-Sposta et al. [10] demonstrated that endurance exercise induced oxidative stress following transient renal impairment and inflammation; however, in humans, the evidence for oxidative stress-mediated tissue damage following exercise remains unclear [9, 11, 12].

This study aimed to investigate the hematological and oxidative changes and tissue damage induced by the marathon race on amateur runners in different thermal conditions: hot and temperate environment. The combination of heat and endurance exercise could promote pronounced physiological response and influence the athletic performance and disease risk.

## 2. Methods

### 2.1. Subjects

Twenty-six Brazilian male endurance runners participated in this study. Volunteers were recruited by e-mail provided by the São Paulo International Marathon Organization (2014 and 2015). After the screening history and medical examination, 71 runners were recruited for the São Paulo International Marathon 2014 (October 19) and 80 runners were recruited for the São Paulo International Marathon 2015 (May 17); however, 26 runners completed both the São Paulo International Marathon 2014 and 2015 and were included in this study. We followed similar experimental procedures and design including the period of blood collection and cardiopulmonary protocol as that of Santos et al. [13].

The exclusion criteria included the use of medication for cardiac, metabolic, pulmonary, or kidney injury and use of alcohol or any kind of drugs and pathologies including systemic arterial hypertension and liver, kidney, metabolic, inflammatory and neoplastic diseases. Subjects were briefed regarding the experimental procedures and the possible risks, and they gave their informed consent before participating, which was approved by the Ethics Committee of Dante Pazzanese Institute of Cardiology, Brazil (permit number: 979/2010), in accordance with the Declaration of Helsinki. Measurements of total body mass (kg), height (cm), and body mass index (BMI, kg/m^2^) were conducted according to the International Society for the Advancement of Kinanthropometry, and the values were expressed as the mean ± standard error of mean (SEM).

### 2.2. Cardiopulmonary Test

Anthropometric parameters were evaluated, and cardiopulmonary exercise test was performed in the same acclimatized room at 21–23°C and 50% relative humidity, 3–21 days before the São Paulo International Marathon 2014 (September 25 to October 16) and 2015 (April 16 to May 14). Functional capacity was assessed by means of cardiopulmonary exercise test (CPET) with expired gas analysis, performed on a treadmill (TEB Apex 200, TEB, São Paulo, Brazil, speed 0–24 km/h, grade 0–35%). A protocol was used, with a starting speed of 8 km/h and grade of 1%; speed was gradually increased by 1 km/h every 1 min. The objective was to achieve fatigue within 8–12 min. Blood pressure was measured with a sphygmomanometer at the commencement of the test. Respiratory gas analysis was performed by the Ergostik (Geratherm, Bad Kissingen, Germany) in breath-by-breath mode.

Tests were considered maximum when at least three of these features were achieved: limiting symptoms/intense physical fatigue, increase in VO_2_ lower than 2.1 mL·kg^−1^·min^−1^ through an increase in speed, attained maximal heart rate, or respiratory quotient higher than 1.1.

### 2.3. Marathon Races

Both races were commenced at 08:00 a.m. and fluid ingestion was allowed ad libitum during the race. Water was provided at every 2-3 km on the running course, sports drinks at 18 and 36 km, and potato or carbohydrate gel at 30 km. The weather parameters at São Paulo International Marathon in 2014 (HE) between 8 a.m. to 2 p.m. were average temperature of 31.4°C, maximum temperature of 35°C, and minimum temperature of 25.8°C; and average relative humidity of 30.4%, maximum relative humidity of 51%, and minimum relative humidity of 26% (National Institute of Meteorology, Ministry of Agriculture, Livestock, and Supply). The weather parameters at the São Paulo International Marathon in 2015 (TE) between 8 a.m. and 2 p.m. were average temperature of 19.8°C, maximum temperature of 22.6°C, and minimum temperature of 16.7°C; and average relative humidity of 72.8%, maximum relative humidity of 86%, and minimum relative humidity of 61% (National Institute of Meteorology, Ministry of Agriculture, Livestock, and Supply). The air quality in the São Paulo International Marathon 2014 was evaluated as low for sulfur dioxide (8.5 *μ*g/m^3^), nitrogen dioxide (9.9 *μ*g/mm^3^), and carbon monoxide (6 ppm) and moderate for particulate matter _10_ (44 *μ*g/m^3^); whereas, in the São Paulo International Marathon 2015, it was evaluated as low for sulfur oxide (7 *μ*g/m^3^), nitrogen dioxide (4.3 *μ*g/mm^3^), carbon monoxide (1.6 ppm), and particulate matter _10_ (19 *μ*g/m^3^).

### 2.4. Blood Collection

Blood samples (30 mL) were collected in vacuum tubes containing an anticoagulant (0.004% EDTA) 24 h before, immediately after, 1 day after, and 3 days after the São Paulo International Marathon in 2014 and 2015. Simultaneously, urine samples were also collected. Biochemical analyses were subsequently performed at the Institute of Physical Activity Sciences and Sports of Cruzeiro do Sul University and at the Clinical Laboratory of Dante Pazzanese Institute of Cardiology. After centrifugation, plasma was aliquoted, frozen, and stored at −80°C for later analysis.

### 2.5. Biochemical Parameters

The biochemical parameters were evaluated with routine-automated methodology in the Clinical Laboratory of Dante Pazzanese Institute of Cardiology immediately after collection. Plasma calcium, magnesium, iron, and bilirubin were performed by colorimetric method; creatinine, C-reactive protein, alanine transaminase, aspartate transaminase, gamma-glutamyl transferase, lactic dehydrogenase, and creatine kinase were determined by kinetic assay; immunological and hematological parameters (hemoglobin, hematocrit, red blood cell distribution width, mean corpuscular volume, mean corpuscular hemoglobin, mean corpuscular hemoglobin concentration, leukocytes, lymphocytes, monocytes, neutrophils, and eosinophils) were assessed by cytochemical/isovolumetric method; ferritin, troponin, proBNP, myoglobin, and creatine kinase-MB were evaluated by chemiluminescence assay; density urine and pH urine were determined by cytometry; and transferrin and alpha-glycoprotein acid were assessed by immunoturbidimetry. Eventually, sodium and potassium were determined by potentiometric ion-selective electrodes.

### 2.6. Determination of Blood Levels for TBARs

For TBAR determination, a slightly modified assay was used [14]. Plasma was mixed thoroughly with butylated hydroxytoluene (BHT, 2%) and trifluoroacetic acid (10%) (4 : 1 : 4) and incubated for 10 min at 4°C; 1,1,3,3-tetramethoxypropane (10^−1^ mM) was used as a positive control. The samples were centrifuged for 15 min at 8000 g. Thereafter, the supernatant was collected and mixed with HCl (25%) and thiobarbituric acid (TBA 1%, dissolved in NaOH 0.05 M) (2 : 1 : 2). The reaction mixture was incubated at 100°C for 30 min, and the absorbance of the supernatant was recorded at 535 nm (SpectraMax Plus, Molecular Devices). A baseline absorbance was considered by running a blank along with all samples during the measurement. The TBAR concentration was calculated using the molar extinction coefficient of malondialdehyde (1.56 × 10^5^).

### 2.7. Superoxide Dismutase 3 Assay

Superoxide dismutase 3 activity was determined by kinetic colorimetric assay on plasma in accordance with the manufacture's protocol (Enzo Life Sciences, NY, USA). The samples and standards were homogenized to SOD Master Mix and xanthine solution was mixed to initiate the reaction. The absorbance readings were recorded at 450 nm at every minute for 10 min at room temperature after 10 s orbital shake prior to the initial read (SpectraMax Plus, Molecular Devices). The slope curve was obtained for each sample and calculated based on the standard curve with sensitivity of 0.1–10 U/L.

### 2.8. Catalase Activity Assay

Catalase activity was determined according to a slightly modified assay [15]. Briefly, 5 *μ*L of plasma samples were added to 200 *μ*L of a dH_2_O and H_2_O_2_ reagent solution (1 : 1000). The absorbance reading of the samples was recorded at 240 nm for 180 s with 30 s intervals at room temperature (SpectraMax 190 Plus, Molecular Devices). The catalase activity calculation was based on the molar extinction coefficient of H_2_O_2_ using an equation: (Δ absorbance/0.0218/mg protein).

### 2.9. Uric Acid Assay

Plasma uric acid was evaluated by enzymatic colorimetric assay and kinetic colorimetric assay in accordance with the manufacture's protocol (Bioclin, MG, Brazil). Standard (6 mg/dL) and plasma samples were mixed with the enzyme reagents and were incubated at 37°C for 5 min. The absorbance readings were recorded at 505 nm (490–540 nm), hitting the zero with the blank (SpectraMax Plus, Molecular Devices). The intensity of the cherry color formed is directly proportional to the concentration of the uric acid in the sample. The uric acid concentration was calculated by the following formula: 6 mg/dL × (sample absorbance/standard absorbance).

### 2.10. Statistical Analyses

Statistical analyses were performed using the Statistical Package for the Social Sciences (IBM SPSS Statistics for Mac, Version 24.0. Armonk, NY, USA). The normality of the data distribution was determined by the Kolmogorov–Smirnov test and rejects the normality. To compare the general and training characteristics in hot and temperate environment, we performed the paired *t*-test (Wilcoxon test). Differences between the steps (before, immediately after, 1 day after, and 3 days after the race) in the hot and temperate environment were tested for significance with the Friedman test with repeated measures and Müller–Dunn's posttest. Spearman correlation was performed to identify the coefficient correlation between the variables using absolute values and relative changes (Δ) compared to baseline levels. Statistical significance was assumed at *p* value < 0.05.

## 3. Results

### 3.1. Sports Performance and Heat Acclimation

The demographic characteristics of the runners and environment parameters are presented in Table 1. Twenty-six athletes finished the São Paulo International Marathon race in the hot environment (HE, mean 31.4°C, humidity 34.3%) and in the temperate environment (TE, mean 19.8°C, humidity 72.8%). The temperature was higher during the 10 days before the race in HE compared to TE, suggesting possible conditions for acclimatization. The training volume, exhaustion speed, time of exhaustion, anaerobic threshold speed, and respiratory compensation point speed after cardiopulmonary test did not differ from 3 to 21 days before the race in 2014 (HE) and 2015 (TE; Table 1). The peak consumption of oxygen (VO_2peak_) was higher in 2014 (HE) compared to 2015 (TE); however, the marathon runner's performance during the race was lower by approximately 13.5%, in accordance with the race time and race pace, in HE compared to TE (Table 1).

### 3.2. Markers of Fluid, Electrolyte Balance, and Kidney Damage

At rest, before the race, we observed that plasma magnesium, sodium concentration, osmolality, and pH urine were greater and plasma potassium and calcium levels were lower for HE indicating fluid and electrolyte imbalance during precompetition (Table 2). The creatinine level also was lower for the HE environment (Table 2). The race time was negatively correlated with urea levels in HE (*r* = 0.5, *p* < 0.01).

In both weathers, marathon race reduces the plasma magnesium concentration (by 20%) and increases the osmolality (by 1.7% to 4%), plasma sodium (by 1% to 3.5%), urea (by 22% to 26%), creatinine (by 50% to 55%), urine density (<1%), hematuria (by 23-fold), and urine WBC (by 6.6-fold), suggesting fluid and electrolyte imbalance and kidney damage. The weight loss was also found to be similar in both 2014 and 2015 marathons (1.8 ± 0.2 kg and 2 ± 0.2 kg in HE and TE, resp.). The osmolality, plasma sodium, and calcium levels were altered 3 days after the race in both the thermal conditions. Moreover, the marathon during HE also promoted elevation of the plasma potassium (by 15%), urine cylinders, and a pronounced physiological response on the osmolality, sodium levels, density urine, urea, hematuria, and urine WBC (Table 2), suggesting exacerbated fluid and electrolyte imbalance, dehydration, and kidney damage in HE. The proteinuria was observed 1 day after the race in HE and 3 days after the race in TE; however, 1 day after the marathon race, creatinine was elevated (by 6%) in TE compared to HE (Table 2).

### 3.3. Markers of Oxidative Damage in the Peripheral Blood

Plasma superoxide dismutase 3 (SOD3) activity and TBARs were lower in HE before and in the recovery period compared to TE. Moreover, we observed a higher catalase activity in HE; however, it was not significant (Table 3). Plasma uric acid and SOD3 activity elevated immediately after the race only in HE. We observed an increase in the TBAR levels immediately after the race in both climates. Three days after the marathon, TBARs and SOD3 activity returned to the baseline levels in both climates. We did not observe any significant alterations in the CAT values in both environments.

### 3.4. Hematological Markers

In both the thermal conditions, erythrocytes, hemoglobin, and hematocrit values reduced after the race and maintained altered 3 days after the race indicating hemolysis and/or hemodilution. In TE, the MCV, MCH, MCHC, and RDW values increased after the race as a consequence of fluid and electrolyte imbalance. In HE, the hemoglobin was higher compared to TE by 3-4% after the race; and the MCV, MCH, MCHC, and RDW values were greater in all periods evaluated compared to TE 1 day after the race (by approximately 1%, 3%, 3%, and 16%, resp.; Table 4).

In HE, we observed correlation between race time and erythrocytes, Hb and Ht, (*r* = 0.45, 0.49, 0.47, 0.47, and 0.57, resp., *p* < 0.01) 1 day after the race. In TE, race time was correlated to unconjugated bilirubin only. In addition, race time was correlated to erythrocytes and Ht-relative changes (∆) 1 day after the race in HE (*r* = 0.44 and 0.4, resp., *p* < 0.05).

After the race, we also demonstrated an increase in the erythropoietin levels (by 16–18%) in both weathers; however, HE promotes a pronounced and earlier response in the erythropoietin levels (Table 4).

### 3.5. Iron Metabolism Markers

Before competition, we observed higher ferritin and transferrin levels (by 17% and 9%, resp.) and lower transferrin saturation (by 8%) in HE. In the recovery period, transferrin remained higher in HE compared to TE (Table 5).

Immediately after the race, we observed an increase in ferritin (by 13% to 23%) and unconjugated and conjugated bilirubin (by 25 to 40%) in both weathers. However, 3 days after the race, we demonstrated a decrease in the iron (by 8% to 25%) and transferrin levels (by approximately 5%) in both weathers, contributing to hemolysis hypothesis after the race. Moreover, HE promoted an elevation in the iron levels 1 day after the race and a reduction in the ferritin level and transferrin saturation 3 days after the race. However, we observed a higher response for the total and unconjugated bilirubin levels in the TE environment as a result of pronounced muscle damage and myoglobin degradation due to higher exercise intensity (Table 5).

### 3.6. Immunological Markers

We observed an elevation in the leukocytes, neutrophils, and monocytes (by approximately 2.5-fold, 3.5-fold, and 1.5-fold, resp.) and a decrease in the lymphocytes after the race by 40%, suggesting exacerbated immune activation after competition. In HE, the lymphocyte and leukocyte alterations were more pronounced 1 day after the race. In the recovery period, 3 days after the race, lymphocytes maintained altered in both weathers, even as leukocytes and eosinophils in TE (Table 6). The eosinophil count decreased after the race only in TE. In HE, the eosinophil count was lower before the race and elevated immediately after the race compared to TE (Table 6).

### 3.7. Muscle Damage Markers and Inflammatory Parameters

In HE, the CRP level and LDH, AST, and ALT activities were lower before and after the race (by approximately 50%) compared to TE and GGT was higher before the race compared to TE. In both weathers, the inflammatory markers, CRP and *α*-1GPA, lactate, and the muscle damage markers, myoglobin, CK, LDH, AST, and ALT increased after the race (by 3 to 4-fold, 1-fold, 2-fold, 5 to 8-fold, 1.8-fold, 23-fold, 2.5-fold, and 1.5-fold, resp.) and maintained altered 3 days after the race, indicating tissue damage. The lactate concentration returned to the basal level 24 h after the race.

The magnitude of changes on the CRP and *α*-1GPA, CK, LDH, AST, and ALT (by 3 to 4-fold, 1-fold, 1.8-fold, 23-fold, 2.5-fold, and 1.5-fold, resp.) was similar in both environments, except for the CK levels, which were 5-fold in HE and 8-fold in TE as a result of the exercise intensity; however, it was not significant (Table 7).

## 4. Discussion

In both environments, the marathon race promotes fluid and electrolyte imbalance, hemolysis, oxidative stress, immune activation, and tissue damage; however, in HE, we demonstrated more pronounced fluid and electrolyte imbalance, renal damage, hemolysis, and immune activation. Moreover, the oxidative stress induced by the marathon in HE appears to be related more to the protein autoxidation than the other oxidative sources.

The loss of water and electrolytes can directly impact the performance and health. The exercise performance is reduced from 7% to 60% by combining the heat stress up to 30°C and dehydration (about >2% body mass) on aerobic exercise over 90 min [5, 16, 17]. The hot and humid environment could also increase the sweat loss and dehydration [4]; however, in our study, the weather was dry and hot. The high humidity in extreme heat stress demonstrates the influence on the performance and cannot be generalized to different climate [2]; hence, the role of humidity on heat stress induced by exercise remains controversial [18, 19]. In the hot environment, we observed impairment of fluid and electrolytic balance before the competition, and markers of hypohydration, as urea and osmolality, after the marathon were accomplished by lower performance in HE, emphasizing the importance of a redoubled attention regarding hydration in competitions in hot environment. In spite of the VO_2_ peak being higher in HE compared to TE, suggesting that the athletes could be more prepared to marathon race in 2014 (HE), the performance on the race was impaired in HE.

The heat acclimatization improves the transpiration capacity, skin blood flow responses, and plasma volume expansion resulting in higher or more stable fluid-electrolyte balance [16, 20]. This heat acclimation improves the ability to reabsorb electrolytes, calcium, copper, magnesium, and sodium specifically, making acclimatized individuals have a lower mineral concentration in sweat, with lower values than 40%, regardless of the amount of transpiration [20]. In our study, before the race, we observed higher levels of plasma sodium, magnesium, and osmolality in HE as a result of hypohydration and/or heat acclimatization increasing the reabsorbing mineral capacity. In HE, we also observed a pronounced electrolyte imbalance as demonstrated by the high levels of sodium and osmolality after the race and lower levels of potassium and calcium 3 days after the race, increasing the risk to hypokalemia and hypernatremia.

Increased sympathetic tone and intravascular volume depletion, may also contribute to reduced renal perfusion increasing the risk of kidney damage during long-distance exercise [10, 21, 22]. The acute kidney injury could induce greater impairment in renal function after cumulative injuries induced by exercise and incomplete recovery of normal renal function during stress [23]. During exercise and heat stress, we also observed both glomerular filtration and renal blood flow reduction, impairing the urine production [5, 24]. Moreover, in the recovery period, we observed impairment in fluid replacement induced by lower capacity to produce urine [5, 24]. In HE, we demonstrated pronounced kidney damage as demonstrated by the elevation of urine density, urea, presence of cylinders, urine hematuria, and urine WBC after the marathon compared to TE. Nevertheless, the creatinine was higher after the race in TE compared to HE. We suggested that the higher creatinine levels in TE could be related to higher CK activity observed in TE (568 U/L versus 481 U/L). A direct relationship between the postrace serum CK and creatinine levels after long-distance running has been reported [23]. Hence, creatinine levels could not reflect the renal damage under stressed conditions; however, a physiological response to the muscle catabolism and/or prerenal component was observed. The proteinuria and hematuria affect 69% and 22% of the marathon runners, respectively [25]. Proteinuria and hematuria may also indicate kidney disorders; however, the transient changes reported after the marathon race are considered as a physiological compensation. The impact of these alterations in long term for permanent renal damage remains unclear [26].

In long-term exercise, excessive fluid and electrolyte loss may lead to hemolysis and promote changes in the renal damage markers. Hemolysis induced by exercise involves mechanical stress from foot strike, red blood cells passing through capillaries in contracting muscles, plasma volume expansion, alterations of erythrocyte membrane proteins, inflammation, and/or oxidative stress [27–29]. Robach et al. [27] suggested that hypernatremia as well inflammation, as we observed in this study, could induce plasma volume expansion and consequently hemolysis. In accordance with other studies [27, 30–32], we demonstrated that hematological changes were induced by long-distance running; moreover, a worsening response in HE could be related to many factors as hypohydration, electrolyte imbalance, and/or oxidative stress. Hemolysis induces iron release and an imbalance in the iron metabolism [32]. In the present study, hemodilution following the race was suggested by decreases in hematocrit, and hemolysis was demonstrated by hematuria, and higher conjugated bilirubin and ferritin levels immediately after the race follow lower erythrocytes, hemoglobin, iron, and transferrin levels 3 days after race. In HE, we also proposed an exacerbated hematological change, reported by higher hematuria, hemoglobin, conjugated bilirubin, and erythropoietin levels. Changes on erythrocytes and Ht were also correlated to race time suggesting that the combination of heat stress and endurance exercise promoted pronounced hematological changes which contribute to impairment of performance. Elevated erythropoietin and MCV observed in HE can indicate the presence of younger cells and erythropoiesis as a result of the heat stress adaptation/response to pronounced hematological changes [27]. Stress hormones such as catecholamine and cortisol also could stimulate enhanced erythropoiesis [33].

Thermal stress also induces oxidative stress, which may contribute to hemolysis and fatigue [34–36]. Exposure of the red blood cells to free oxygen radicals may promote hemoglobin damage and induce protein degradation, lipid peroxidation, and hemolysis; however, oxidative stress may be more attributed to hypohydration than heat during exercise [37]. The hyperthermia inactivates antioxidative enzymes causing deleterious effects on lipoperoxidation and redox status [34]. In fact, in HE, we observed lower SOD3 activity compared to TE before and in the recovery period; however, we observed an increase in the SOD3 activity after the marathon race in HE reaching the same SOD activity observed in TE environment. A previous study also reported a postexercise antioxidant response in hot and humid environment by elevation of the catalase activity after a treadmill exercise of 45 min at 75–80% of maximal oxygen uptake in well-trained athletes [36]. In HE, we also reported a reduced lipoperoxidation compared to TE; however, a greater uric acid production suggested pronounced purine and protein degradation in erythrocytes or muscle and diffusion of hypoxanthine and uric acid into the bloodstream. Hyperuricemia may induce glomerular hypertension, whereas the increased urinary uric acid may directly injure the renal tubules as a result of exercise and heat stress [24]. Davies and Goldberg [31, 38] described that lipoperoxidation and protein oxidation imply different processes *in vivo* and that protein oxidation occurs more quickly than lipoperoxidation. Maughan et al. [39] demonstrated that lipoperoxidation initiated by free radicals was associated with the muscle damage and its respective markers. In accordance to this study, we demonstrated that TBARs were accomplished by higher levels of muscle markers CK, LDH, ALT, and AST in TE, agreeing that lipoperoxidation initiated by free radicals was associated with muscle damage.

In contrast with our study, after 4 weeks of high-intensity interval training, we observed an increase in protein carbonyls with no changes in TBARs induced by exercise in TE and an increase in TBARs with no changes in protein carbonyls in HE [40].

Inflammatory state also has been associated with oxidative stress and tissue damage [10]. Exercise-induced muscle damage is accompanied by exacerbated immune activation and inflammatory mediators [13]. In this study, the marathon race promoted immune activation and inflammation in both environments. In HE, the leukocytosis and lymphopenia were also more pronounced 1 day after the race. A previous study demonstrated a higher proinflammatory response (MCP-1) following an anti-inflammatory response (IL-6) after the treadmill test in heat stress that could contribute to the understanding of the pronounced inflammatory response (leukocytosis) followed by anti-inflammatory response (lymphopenia) induced by the marathon and the lower levels of CRP in HE [36], in spite of half marathon not demonstrating inflammatory or immunological changes in hot and humid environment [41].

In HE, the enzymes associated to muscle/hepatic damage (LDH, AST, and ALT) and CRP were lower in all periods evaluated suggesting lower exercise or training intensity or inactivation of the metabolic enzyme activity by heat stress. Nevertheless, the magnitude of the change induced long-distance exercise was greater in HE only for the CK levels (5-fold versus 7.5-fold, *p* > 0.05), but was not significant. The CK levels usually maintained altered 3–7 days after intense/prolonged exercise [42], which could explain the values above the reference value in TE. In this study, 13 athletes were above the reference value in TE and 9 athletes were above the reference value in HE before the race indicating less than 1 week of rest before race.

Thus, long-term exercise accomplished by heat stress may compromise the performance by hematological alterations due to fluid and electrolyte imbalance and purine or protein oxidation in erythrocytes, leading to renal damage and immune activation. Nonetheless, we suggested that the impairment on the performance in HE was not associated to the muscle damage and lipoperoxidation. Protein autoxidation and fluid and electrolyte imbalance may be an important factor responsible for hemolysis and impaired performance after long-term exercise in HE.

## Figures and Tables

**Table 1:** General and training characteristics of the marathon runners and environment parameters in the hot and temperate environment.

	Hot environment	Temperate environment
Age (years)	42 ± 2	43 ± 2
Height (m)	1.73 ± 0.01	1.73 ± 0.01
Body mass (kg)	75 ± 2	75 ± 2
BMI (kg/m^2^)	25.2 ± 0.2	25.1 ± 0.2
Training experience (years)	6.11 ± 0.22	6.69 ± 0.80
Training volume (km/week)	55.2 ± 3.2	52.03 ± 2.39
Time of exhaustion (min)	10.9 ± 0.4	11.1 ± 0.5
VO_2peak_ (mL·kg^−1^·min^−1^)	58.6 ± 1.2^∗^	54.6 ± 1.2
Exhaustion speed (km/h)	18.3 ± 0.3	18.4 ± 0.3
AT speed (km/h)	9.3 ± 0.2	9.3 ± 0.2
RCP speed (km/h)	16.6 ± 0.4	16.5 ± 0.5
Race time (min)	276 ± 8^∗^	243 ± 11
Race pace (min/km)	6.54 ± 0.18^∗^	5.75 ± 0.26
Temperature (°C) 10 days before the race	23.4 ± 0.6^∗^	16.3 ± 0.2
Temperature (°C) at race	31.4 ± 0.6^∗^	19.8 ± 0.2
Humidity at race (%)	30.4 ± 7.7^∗^	72.8 ± 7.2

BMI: body mass index; AT: anaerobic threshold; RCP: respiratory compensation point; VO_2peak_: peak consumption of oxygen. The values presented are the mean ± standard error of mean (SEM) of 26 runners. ^∗^*p* < 0.05 for comparison between hot and temperate environment.

**Table 2:** Fluid, electrolyte balance, and renal function after the marathon race in the temperate and hot environment.

	Reference values	Before	Immediately after	1 day after	3 days after
Sodium (mMol/L)	137–145				
HE		142 ± 0.4^##^	147 ± 0.5^∗∗^^##^	142 ± 0.4^#^	143 ± 0.3^∗^^#^
TE		139 ± 0.3	140 ± 0.4	140 ± 0.3^∗^	141 ± 0.3^∗∗^
Magnesium (mg/dL)	1.7–2.5				
HE		2.5 ± 0.04^#^	2 ± 0.05^∗∗^^#^	2.2 ± 0.03^∗∗^	2.4 ± 0.03^∗^^#^
TE		2.3 ± 0.1	1.9 ± 0.1^∗∗^	2.2 ± 0.1^∗^	2.3 ± 0.1
Osmol (mOsm/L)	275–295				
HE		296 ± 1^##^	309 ± 1^∗∗^^##^	298 ± 1^∗∗^^##^	297 ± 1^##^
TE		289 ± 1	294 ± 1^∗∗^	293 ± 1^∗∗^	293 ± 1^∗∗^
Potassium (mMol/L)	3.5–5.6				
HE		3.9 ± 0.05^##^	4.5 ± 0.1^∗∗^	4.4 ± 0.1^∗∗^	4.3 ± 0.1^∗∗^^#^
TE		4.5 ± 0.05	4.4 ± 0.1	4.5 ± 0.1	4.5 ± 0.1
Calcium (mg/dL)	8.4–11				
HE		9.3 ± 0.1^##^	9.3 ± 0.1^##^	9.1 ± 0.1^##^	8.9 ± 0.1^∗^^##^
TE		9.6 ± 0.1	9.8 ± 0.1^∗^	9.6 ± 0.1	9.5 ± 0.1^∗^
Creatinine (mg/dL)	0.7–1.3				
HE		0.9 ± 0.02^##^	1.4 ± 0.05^∗∗^^#^	1 ± 0.03^∗^	0.9 ± 0.02^#^
TE		1 ± 0.02	1.5 ± 0.1^∗∗^	1 ± 0.03	1 ± 0.03
Urea (mg/dL)	<40				
HE		40 ± 1	49 ± 2^∗∗^	49 ± 2^∗∗^^#^	38 ± 1^#^
TE		38 ± 1	48 ± 1^∗∗^	46 ± 2^∗∗^	35 ± 2
UD (kg/m^3^)	1005–1030				
HE		1019 ± 2	1025 ± 1^∗^^#^	1022 ± 1^#^	1021 ± 2^#^
TE		1016 ± 1	1019 ± 1^∗^	1013 ± 1^∗^	1014 ± 1
Urine pH	5-6				
HE		6 ± 0.1^#^	6 ± 0.1^##^	6 ± 0.1^##^	6 ± 0.1^#^
TE		6 ± 0.2	5 ± 0.1^∗^	5 ± 0.1	6 ± 0.1^∗^
Urine WBC (cell/mL)	0–10,000				
HE		1638 ± 314	10,828 ± 2170^∗∗^^#^	4589 ± 569^∗∗^^#^	2778 ± 755
TE		2310 ± 598	4828 ± 1185^∗^	2071 ± 230	2250 ± 444
Hematuria (cell/mL)	0–10,000				
HE		3259 ± 1539	76,017 ± 36,939^∗∗^	3679 ± 736^#^	4259 ± 1409^#^
TE		1241 ± 146	44,241 ± 13,538^∗^	4679 ± 2070	2500 ± 878
Urine cylinders (units/mL)	0				
HE		0 ± 0	463 ± 144^∗^^#^	0 ± 0	0 ± 0
TE		69 ± 48	35 ± 35	107 ± 79	0 ± 0
Proteinuria (mg/dL)	0				
HE		3 ± 2	9 ± 5	1 ± 1^#^	0 ± 0
TE		3 ± 2	1 ± 1	7 ± 3	2 ± 2

WBC: white blood cells; UD: urine density; Osmol: osmolality; HE: hot environment; TE: temperate environment. The values presented are the mean ± standard error of mean (SEM) of 26 runners. ^∗^*p* < 0.05 versus before race; ^∗∗^*p* < 0.0001 versus before race; ^#^*p* < 0.05; and ^##^*p* < 0.0001 versus temperate environment.

**Table 3:** Redox status after marathon race in temperate and hot environment.

	Before	Immediately after	1 day after	3 days after
Uric acid (mg/dL)				
HE	5.4 ± 0.1	6.6 ± 0.3^∗∗^	5.7 ± 0.2	5.6 ± 0.3
TE	5.9 ± 0.2	6.3 ± 0.2	6.1 ± 0.2	6.1 ± 0.3
SOD3 (U/mL)				
HE	2.6 ± 0.2^#^	3.2 ± 0.4^∗^	2.4 ± 0.3^##^	2.6 ± 0.2^#^
TE	3.3 ± 0.1	3.1 ± 0.1	3.7 ± 0.5	3.1 ± 0.2
TBARs (nMol/mL)				
HE	2.4 ± 0.05^#^	4.1 ± 0.01^∗∗^^#^	3.2 ± 0.05^∗^^#^	2.6 ± 0.2^#^
TE	3.1 ± 0.2	4.6 ± 0.1^∗∗^	3.9 ± 0.1^∗^	3.3 ± 0.02
CAT (U/mL)				
HE	87.9 ± 8	63.2 ± 9	70.5 ± 8	73.7 ± 8
TE	72.8 ± 9	49.0 ± 5	49.8 ± 6	59.2 ± 5

SOD: superoxide dismutase; CAT: catalase; TBARs: thiobarbituric acid reactive substances; HE: hot environment; TE: temperate environment. The values are presented as mean ± standard error of mean (SEM) of 26 runners. ^∗^*p* < 0.05 versus before the race; ^∗∗^*p* < 0.0001 versus before the race; ^#^*p* < 0.05; and ^##^*p* < 0.0001 versus temperate environment.

**Table 4:** Hematological parameters after the marathon race in the temperate and hot environments.

	Reference value	Before	Immediately after	1 day after	3 days after
Erythrocytes (10^6/mm^3^)	4.5–5.5				
HE		5.2 ± 0.1	5.2 ± 0.1	4.9 ± 0.1^∗∗^	4.9 ± 0.1^∗∗^
TE		5.3 ± 0.1	5.1 ± 0.1^∗^	4.9 ± 0.1^∗∗^	4.9 ± 0.1^∗∗^
Hemoglobin (g/dL)	13–17				
HE		15.4 ± 0.2	15.6 ± 0.2^#^	14.6 ± 0.2^∗∗^^#^	14.5 ± 0.1^∗∗^
TE		15.2 ± 0.2	15.0 ± 0.2	14.2 ± 0.2^∗∗^	14.5 ± 0.1^∗∗^
Hematocrit (%)	40–50				
HE		47 ± 0.5	46 ± 0.5^∗^	44 ± 0.5^∗∗^	45 ± 0.5^∗∗^
TE		47 ± 0.5	46 ± 0.5^∗^	44 ± 0.7^∗∗^	45 ± 0.4^∗∗^
Erythropoietin (mU/mL)	4.3–29				
HE		12 ± 1	14 ± 1^∗^^#^	14 ± 1	14 ± 1^∗^^#^
TE		11 ± 1	12 ± 1	12 ± 1	13 ± 1^∗^
MCV (fL)	80–100				
HE		90 ± 1^#^	89 ± 1^∗^	90 ± 1^#^	89 ± 2
TE		89 ± 1	89 ± 1	89 ± 1	91 ± 1^∗^
MCH (pg)	27–32				
HE		30 ± 0.2^##^	30 ± 0.3^∗^^##^	28 ± 1^#^	30 ± 0.2^#^
TE		29 ± 0.2	29 ± 0.2^∗^	29 ± 0.2	29 ± 0.3^∗^
MCHC (g/dL)	31.5–36				
HE		33 ± 0.1^#^	34 ± 0.2^∗^^#^	33 ± 0.2^#^	33 ± 0.1^#^
TE		32 ± 0.1	33 ± 0.2^∗^	32 ± 0.1	32 ± 0.2^∗^
RDW (%)	11.9–15.4				
HE		14 ± 0.1^##^	14 ± 0.1^##^	14 ± 0.1^##^	14 ± 0.1^##^
TE		12 ± 0.1	13 ± 0.1^∗∗^	13 ± 0.1^∗∗^	13 ± 0.1^∗∗^

MCV: mean corpuscular volume; MCH: mean corpuscular hemoglobin; MCHC: mean corpuscular hemoglobin concentration; RDW: red cell distribution width; HE: hot environment; TE: temperate environment. The values are presented as mean ± standard error of mean (SEM) of 26 runners. ^∗^*p* < 0.05 versus before the race; ^∗∗^*p* < 0.0001 versus before the race; ^#^*p* < 0.05; and ^##^*p* < 0.0001 versus temperate environment.

**Table 5:** Iron metabolism and hemolysis biomarkers after marathon race in the temperate and hot environments.

	Reference value	Before	Immediately after	1 day after	3 days after
Iron (*μ*g/L)	60–150				
HE		110 ± 8	120 ± 7	142 ± 10^∗^	90 ± 6^∗^
TE		120 ± 9	126 ± 9	130 ± 8	89 ± 6^∗^
Ferritin (ng/L)	23–336.2				
HE		172 ± 23^#^	194 ± 25^∗^	186 ± 24^∗^^#^	147 ± 20^∗∗^
TE		147 ± 20	181 ± 25^∗∗^	169 ± 21^∗∗^	152 ± 17
Transferrin (mg/dL)	170–340				
HE		280 ± 4^##^	277 ± 4	277 ± 4^#^	266 ± 3^∗^^##^
TE		257 ± 6	275 ± 7^∗∗^	248 ± 5	242 ± 4^∗∗^
Transferrin saturation (%)	20–50				
HE		39 ± 3^#^	43 ± 3	51 ± 3^∗^	34 ± 2^∗^
TE		47 ± 4	45 ± 3	54 ± 4^∗^	38 ± 3
Total bilirubin (mg/dL)	0.1–1.2				
HE		0.8 ± 0.1	0.9 ± 0.1^#^	1.1 ± 0.1^∗^	0.7 ± 0.05
TE		0.9 ± 0.1	1.1 ± 0.1^∗^	1.1 ± 0.1^∗^	0.8 ± 0.1^∗^
Unconjugated bilirubin (mg/dL)	0.1–1.2				
HE		0.4 ± 0.04	0.5 ± 0.06^∗^^#^	0.6 ± 0.1^∗^^#^	0.4 ± 0.03
TE		0.5 ± 0.1	0.7 ± 0.1^∗^	0.8 ± 0.1^∗^	0.4 ± 0.1^∗^
Conjugated bilirubin (mg/dL)	<0.3				
HE		0.3 ± 0.02	0.4 ± 0.03^∗^	0.4 ± 0.04^∗^^#^	0.3 ± 0.02
TE		0.3 ± 0.02	0.4 ± 0.03^∗^	0.3 ± 0.01	0.3 ± 0.02

The values are presented as mean ± standard error of mean (SEM) of 26 runners. HE: hot environment; TE: temperate environment. ^∗^*p* < 0.05 versus before the race; ^∗∗^*p* < 0.0001 versus before the race; ^#^*p* < 0.05; and ^##^*p* < 0.0001 versus temperate environment.

**Table 6:** Immunological parameters after the marathon race in the temperate and hot environments.

	Reference value	Before	Immediately after	1 day after	3 days after
Leukocytes (mil/mm^3^)	4.5–11				
HE		6.1 ± 0.4	14.4 ± 0.6^∗∗^	7.6 ± 0.4^∗^^#^	5.8 ± 0.2
TE		6.0 ± 0.2	15.3 ± 0.6^∗∗^	7.1 ± 0.3^∗∗^	5.4 ± 0.2^∗^
Lymphocytes (mil/mm^3^)	1–3.8				
HE		1.8 ± 0.1	1.1 ± 0.1^∗∗^^#^	2 ± 0.1^#^	1.7 ± 0.1^∗^
TE		2 ± 0.1	1.3 ± 0.1^∗∗^	1.8 ± 0.1	1.7 ± 0.1^∗^
Neutrophils (mil/mm^3^)	1.8–7.7				
HE		3.6 ± 0.3	12.4 ± 0.5^∗∗^	4.9 ± 0.3^∗∗^	3.4 ± 0.2
TE		3.4 ± 0.2	13.2 ± 0.6^∗∗^	4.6 ± 0.2^∗∗^	3.1 ± 0.1
Monocytes (mil/mm^3^)	0–0.8				
HE		0.41 ± 0.03	0.60 ± 0.04^∗∗^	0.49 ± 0.03^∗^	0.39 ± 0.02
TE		0.37 ± 0.02	0.71 ± 0.07^∗∗^	0.47 ± 0.03^∗∗^	0.36 ± 0.02
Eosinophils (mil/mm^3^)	0–0.45				
HE		0.16 ± 0.02^#^	0.19 ± 0.02^##^	0.15 ± 0.03	0.16 ± 0.02
TE		0.23 ± 0.04	0.02 ± 0.01^∗∗^	0.17 ± 0.03^∗∗^	0.18 ± 0.03^∗^

The values are presented as mean ± standard error of mean (SEM) of 26 runners. HE: hot environment; TE: temperate environment. ^∗^*p* < 0.05 versus before the race; ^∗∗^*p* < 0.0001 versus before the race; ^#^*p* < 0.05; and ^##^*p* < 0.0001 versus temperate environment.

**Table 7:** Inflammatory mediators and markers of muscle damage after the marathon race in the temperate and hot environments.

	RV	Before	Immediately after	1 day after	3 days after
CRP (mg/dL)	<0.8				
HE		0.4 ± 0.01^##^	0.4 ± 0.01^##^	1.7 ± 0.2^∗∗^^#^	0.6 ± 0.05^∗∗^^##^
TE		0.8 ± 0.05	0.6 ± 0.05^∗^	2.5 ± 0.2^∗∗^	1.3 ± 0.1^∗∗^
*α*-1GPA (mg/dL)	50–120				
HE		66 ± 3	77 ± 3^∗∗^	77 ± 2^∗∗^	72 ± 3^∗^
TE		69 ± 3	75 ± 3^∗∗^	77 ± 3^∗∗^	78 ± 3^∗^
Myoglobin (ng/mL)	<106				
HE		44 ± 6	1016 ± 126^∗∗^	154 ± 20^∗∗^	84 ± 19^∗∗^
TE		46 ± 7	1059 ± 120^∗∗^	184 ± 26^∗∗^	96 ± 35^∗^
CK (U/L)	<171				
HE		190 ± 28	481 ± 56^∗∗^	1427 ± 246^∗∗^	906 ± 381^∗^
TE		393 ± 155	568 ± 78^∗∗^	1828 ± 283^∗∗^	1231 ± 497^∗^
LDH (U/L)	120–246				
HE		252 ± 12^##^	427 ± 33^∗∗^^##^	290 ± 19^∗^^##^	284 ± 20^##^
TE		500 ± 23	934 ± 37^∗∗^	669 ± 27^∗∗^	625 ± 32^∗∗^
Lactate (mmol/L)	0.5–1.6				
HE		1.1 ± 0.1	2.4 ± 0.2^∗∗^	1.2 ± 0.1	0.9 ± 0.1
TE		1.2 ± 0.1	2.8 ± 0.2^∗∗^	0.9 ± 0.1	1.0 ± 0.1
AST (U/L)	<50				
HE		28 ± 1^#^	37 ± 2^∗∗^^#^	75 ± 10^∗∗^	48 ± 7^∗^^#^
TE		37 ± 3	52 ± 4^∗∗^	85 ± 7^∗∗^	64 ± 7^∗∗^
ALT (U/L)	<50				
HE		24 ± 2^##^	25 ± 2^##^	29 ± 3^∗^^##^	34 ± 4^∗^^#^
TE		39 ± 2	42 ± 2^∗^	48 ± 2^∗∗^	52 ± 2^∗∗^
GGT (U/L)	<73				
HE		34 ± 4^#^	33 ± 3	31 ± 3^∗^	30 ± 3^∗^
TE		30 ± 3	32 ± 3^∗^	29 ± 3	28 ± 3^∗^

RV: reference value; *α*-1GPA: alpha 1 acid glycoprotein; CRP: C-reactive protein; LDH: lactate dehydrogenase; CK: creatine kinase; ALT: alanine transaminase; AST: aspartate transaminase; GGT: gamma-glutamyl transferase; HE: hot environment; TE: temperate environment. The values are presented as mean ± standard error of mean (SEM) of 26 runners. ^∗^*p* < 0.05 versus before the race; ^∗∗^*p* < 0.0001 versus before the race; ^#^*p* < 0.05; and ^##^*p* < 0.0001 versus temperate environment.
